# 

**Published:** 1888-11

**Authors:** 


### TABLE OF CONTENTS.

### Original and Selected Articles.

The surgery of gummatous growths of the nasal cavities, by A. G. Hobbs, M. D., Prof. of Opthalmology, Otology, Rhino-Laryngology in Southern Medical College, Atlanta. 401
A large vesico-vaginal fistula, with everted bladder into vaginal canal. Conception reaching four and a half or five months gestion—operation, etc., by K. P. Moore, M. D., of Ga. 405
Epistaxis and hemorrhage from a wound in the superior coronary successfully treated by compression upon the artery, by Frank B. Humphreys, M. D., Hawkins, Tex. 410
A plea for a more amicable relationship and greater sociability between physicians, by T. B. Greenley, M. D. 414
Typhoid Fever; cold water enemata in cholera infantum 417

### Abstracts and Gleanings.

Calomel; carbolic acid in hemorrhoids 418
Hot pack in pneumonia 419
A valuable formula in menstrual irregularities 420
Emergency case; goitre; diabetes—a new drug in its treatment 421
Goitre—gonorrhœa; a valuable remedy for chronic diarrhœa 422
Arrest of hemorrhage from wounds of the palm of the hand; aphrodisiac; Battey vs. Lawson Tait 423
External application of sulphur in sciatic neuralgia 424
Antiseptic method of treating burns and scalds; carbuncle; diphtheria 425
New operation for incontinence of urine in women; pilocarpine in diphtheria; strychnia in dyspepsia 426
The treatment of chronic diarrhœa by talc; hydrotherapy during menstruation; idoform poisoning 427
A remarkable case; acute tonsillitis in children; digestive disorders of children; dose of aconitia—a warning 428
Chronic syphilitic salivation; painless destruction of nævia; use of boracic acid; a vaginal tampon 429
Antipyrin as an agalactic; transplantation of skin from a corpse; the best method; reduction of paraphymosis; quassi in drunkenness; methylal in delirium tremens 430

### Scientific Items.

Psychic effects of hasheesh; detection of typhoid bacilli; fire and water proof paper 431
Invention of the circular saw; photographs of the eye; to color iron blue; monitors engineering inventions 432

### Practical Notes and Formulæ.

To abort furunculus; neuralgia; muriate of ammonia in myalgia; to abort a boil; earache 433
Bromidrosis; treatment of rheumatism; eczema of the head; habitual constipation; leucorrhœa; worms 434

### Editorials and Miscellaneous.

Editorial brevities 435
Write for your journal; he comes back; tonsilitis; the American exhibit to be made at the Paris World's Fair 436
The Homiletic Review; Dr. Mackenzie's book 437
Books and pamphlets received 438
Special notes 440

